# Dataset for measured viscosity of Polyalpha-Olefin- boron nitride nanofluids

**DOI:** 10.1016/j.dib.2021.106881

**Published:** 2021-02-15

**Authors:** Ahmad K. Sleiti

**Affiliations:** Department of Mechanical & Industrial Engineering, College of Engineering, Qatar University, Qatar

**Keywords:** Nanofluids, Thermophysical properties, Polyalpha-olefin, PAO, Boron nitride, Viscosity, Lubricant

## Abstract

Datasets of measured viscosity of Polyalpha-Olefin- boron nitride (PAO/hBN) nanofluids are reported. An AR-G2 rheometer (from TA Instruments) experimental setup is used for measuring the rheological property of PAO/hBN nanofluids, which is a combined motor and transducer (CMT) instrument. The test fluid sample size is approximately 1.5 ml and the tests were conducted over a temperature range of the tested fluids from − 20 °C to 70 °C by a water circulator chamber. The dataset includes measured viscosities as a function of the BN volumetric concentration (*ϕ*) of 0, 0.6 and 1%. Two sets of viscosity measurements are conducted insuring the thermal equilibrium conditions are reached for all experiments. In set (1), the viscosity is measured at intervals of 10 °C by fixing the temperature at each interval (at −20, −10, 0, 10, 20, 30, 40, 50, 60 and 70 °C), while the shear stress and shear rate are varied. In set (2), the temperature is varied from −20 °C to 70 °C at intervals of 0.5 °C, while the shear stress is fixed and the shear rate is varied accordingly. Set (1) is designed to verify whether the fluids are Newtonian or not and set (2) is designed to derive correlations for the viscosity as a function of temperature. Several characteristics data are recorded including rotational speed of the spindle (RPM), torque, viscosity (Pa- s), shear stress (Pa), shear strain rate (1/s) and temperature (°C). The reuse potential of the dataset includes calculating Reynolds number for further flow studies; heat transfer performance studies of nanofluids; lubrication and lubricants’ development studies and characteristics of Newtonian and non-Newtonian fluids. The dataset reported here were used (but not published) in the article published by the author in [1] (https://doi.org/10.1016/j.csite.2020.100776).

## Specifications Table

**Table:** 

Subject	Energy (Nanofluids)
Specific subject area	Nanofluids for heat transfer and lubrication; viscosity measurements
Type of data	Tables Figures
How data were acquired	The data were acquired using AR-G2 rheometer (from TA Instruments, 2005) experimental setup for measuring the rheological property of PAO/hBN nanofluids, which is a combined motor and transducer (CMT) instrument. The raw data were exported to Excel spreadsheets.
Data format	Raw and Filtered Data
Parameters for data collection	The two sets of viscosity measurements data are collected at intervals of 10 °C and at intervals of 0.5 °C insuring the thermal equilibrium conditions are reached for all experiments
Description of data collection	The data were collected using the measurement method described above. The data were saved and exported to Excel spreadsheets for further organizing.
Data source location	Institution: Qatar University City/Town/Region: Doha Country: Qatar
Data accessibility	The data are hosted ‘With the article’.
Related research article	The data article is related to the following research article: A.K. Sleiti, Heat transfer measurements of Polyalpha-Olefin- boron nitride nanofluids for thermal management and lubrication applications, Case Stud. Therm. Eng. 22 (2020) 100,776. https://doi.org/10.1016/j.csite.2020.100776.

## Value of the Data

• The data are important and useful because the reuse potential of the dataset includes calculating Reynolds number for further flow studies; heat transfer performance studies of nanofluids; lubrication and lubricants’ development studies [2], [3], [4], [5] and characteristics of Newtonian and non-Newtonian fluids [6].
• The data can benefit engineers, researchers and scientists working in the fields of energy, thermofluids, power systems, energy storage, materials, cooling, heating and lubrication.
• The data can be used/reused for further insights and development of experiments by extrapolating the data to more ranges of temperature and concentration and testing new sets of PAO based nanofluids.
• The artificial neural networks are high performance predictive tools. The data of the present study can be used for comparative analysis studies of the predictive performance of such artificial neural networks that can be developed using the experimental viscosity results of the nanofluids of the present study.

## 1 Data Description

**Fig. 1:**
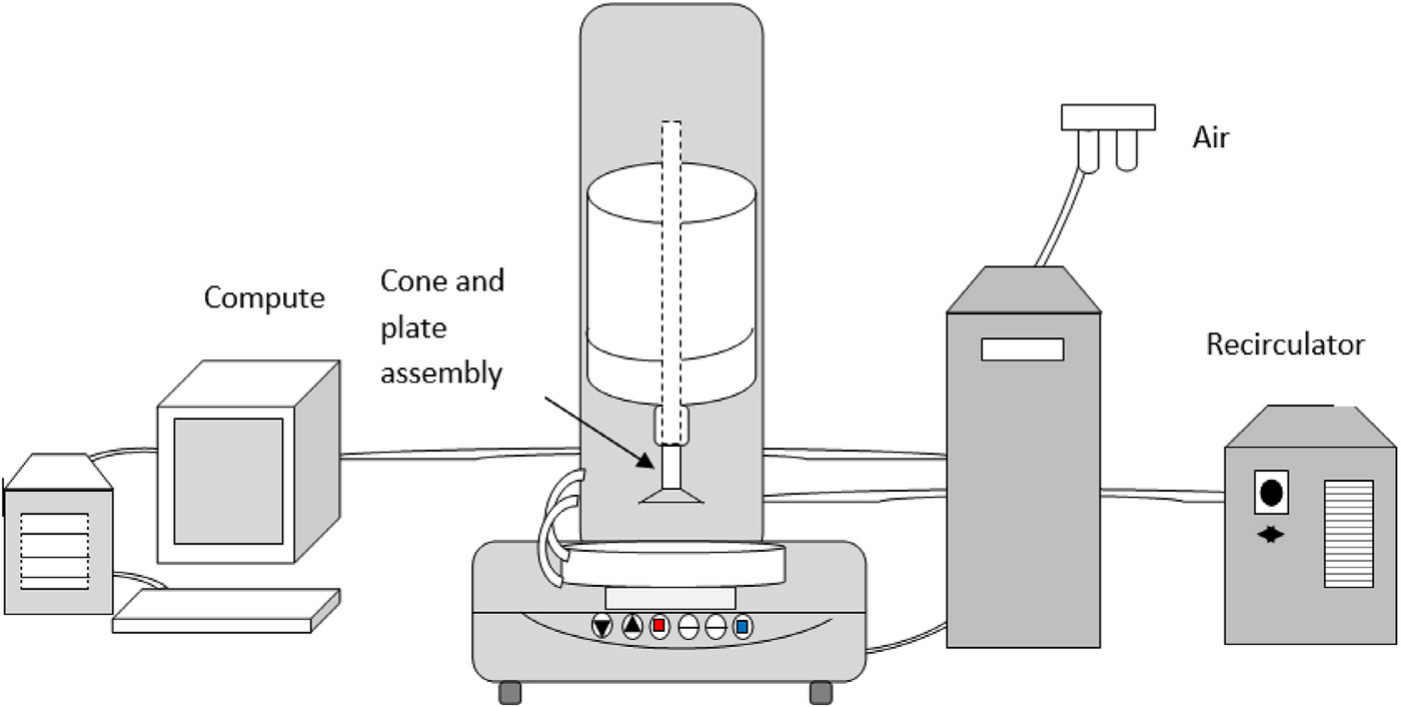
Viscosity measurement of nanofluids - Experimental setup schematic.

The data provided in this article are related to the published article in [1]. Fig. 1 shows schematic for the experimental setup used for the viscosity measurements of the pure PAO base fluid and for the PAO/hBN nanofluids. The AR-G2 rheometer from TA Instruments is used. The constraint on the low torque performance of such an instrument is the friction between the rotating and the stationary components. The temperature of the tested fluids is controlled between −20 °C to 70 °C by a water circulator chamber. Different data were taken including rotational speed of the spindle (RPM), torque, viscosity (Pa-s), shear stress (Pa), shear strain rate (1/s) and temperature (°C).

**Fig. 2:**
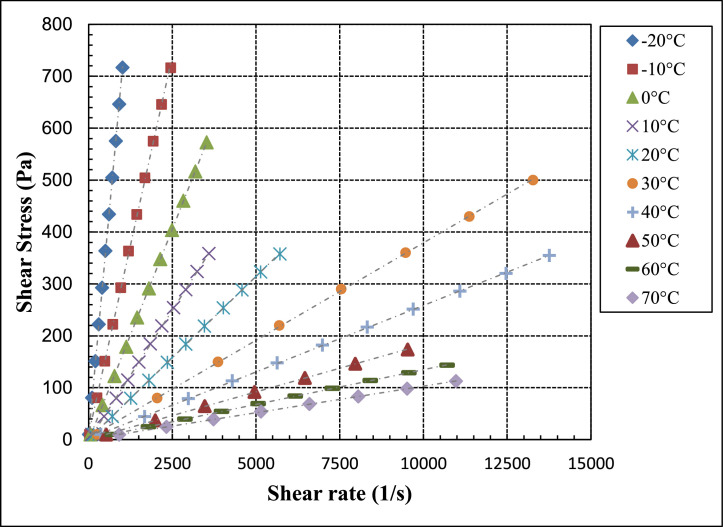
Shear stress versus shear strain for pure PAO base fluid over a temperature range from negative 20 to 70 °C.

Shear stress, shear rate, and viscosity data are provided in Tables 1, 3 and 5 (data set (1)) for pure PAO base fluids (*ϕ* = 0%), for PAO/hBN nanofluids with BN particle concentration, *ϕ* = 0.6%, and 1.0%, respectively as a function of temperature from negative 20 to 70 °C at intervals of 10 °C. This set of data is used to characterize the fluids in terms of Newtonian or non-Newtonian fluids. Figs. 2, 4 and 6 illustrate the data of Tables 1, 3 and 5, respectively in terms of shear stress versus shear strain for pure PAO base fluids (*ϕ* = 0%), for PAO/hBN nanofluids with BN particle concentration, *ϕ* = 0.6%, and 1.0%, respectively over the full temperature range from negative 20 to 70 °C.

**Table 1:** Raw data for pure PAO base fluids (*ϕ* = 0) as a function of temperature at fixed temperature intervals and varied shear and shear rate. This set of data is used to verify whether the fluid is Newtonian or non-Newtonian.

Shear stress	Shear rate	Viscosity	Temperature
Pa	1/s	Pa.s	°C
9.995	13.44	0.7438	−20
80.67	110.3	0.7314	−20
151.3	208.3	0.7266	−20
222	308.8	0.719	−20
292.7	406	0.7209	−20
363.4	509.5	0.7132	−20
434.1	612.2	0.709	−20
504.7	709.2	0.7117	−20
575.4	816.3	0.7049	−20
646.1	917.7	0.704	−20
716.8	1017	0.7046	−20
787.4	1135	0.6939	−20
858.1	1245	0.6893	−20
928.8	1357	0.6847	−20
999.4	1455	0.6867	−20
9.988	31.49	0.3172	−10
80.62	257.1	0.3136	−10
151.2	487.5	0.3102	−10
221.9	723.8	0.3065	−10
292.5	961.5	0.3042	−10
363.1	1199	0.3029	−10
433.7	1443	0.3005	−10
504.4	1686	0.2991	−10
575	1933	0.2975	−10
645.6	2186	0.2954	−10
716.2	2453	0.292	−10
786.8	2720	0.2893	−10
857.4	2986	0.2872	−10
928	3268	0.284	−10
998.6	3550	0.2813	−10
9.976	62.7	0.1591	3.00E-03
66.27	419.1	0.1581	−2.00E-03
122.6	779.3	0.1573	−2.00E-03
178.9	1130	0.1582	3.00E-03
235.2	1454	0.1618	3.00E-03
291.5	1805	0.1614	−6.00E-03
347.7	2146	0.1621	3.00E-03
404	2497	0.1618	0
460.3	2834	0.1624	0
516.6	3188	0.1621	−2.00E-03
572.9	3530	0.1623	0
629.2	3879	0.1622	0
685.5	4253	0.1612	3.00E-03
741.8	4646	0.1597	−2.00E-03
798.1	5038	0.1584	−6.00E-03
9.96	103.8	0.09593	10
44.82	469.6	0.09544	10
79.68	831.3	0.09585	10
114.6	1177	0.09731	10
149.4	1509	0.09901	10
184.3	1845	0.0999	10
219.2	2192	0.09997	10
254	2544	0.09987	10
288.9	2906	0.09942	10
323.8	3242	0.09986	10
358.6	3595	0.09975	10
393.5	3936	0.09998	10
428.4	4278	0.1001	10
463.2	4633	0.09998	10
498.1	4961	0.1004	10
9.941	155.1	0.0641	20
44.73	709.3	0.06306	20
79.52	1264	0.06289	20
114.3	1806	0.06329	20
149.1	2357	0.06327	20
183.9	2903	0.06335	20
218.7	3461	0.06317	20
253.5	4025	0.06297	20
288.2	4581	0.06293	20
323	5139	0.06286	20
357.8	5715	0.06261	20
9.903	253.4	0.03908	30
79.93	2048	0.03902	30
150	3865	0.0388	30
220	5694	0.03863	30
290	7549	0.03841	30
360	9465	0.03803	30
429.9	11,370	0.03783	30
499.9	13,270	0.03768	30
569.9	15,180	0.03755	30
9.857	373.6	0.02639	40
44.36	1676	0.02647	40
78.86	2984	0.02642	40
113.4	4296	0.02638	40
147.8	5635	0.02624	40
182.3	6985	0.0261	40
216.8	8327	0.02604	40
251.3	9700	0.0259	40
285.8	11,090	0.02577	40
320.2	12,470	0.02568	40
354.7	13,760	0.02579	40
9.797	530.4	0.01847	50
37.09	1998	0.01856	50
64.39	3472	0.01854	50
91.67	4956	0.0185	50
119	6463	0.01841	50
146.2	7969	0.01835	50
173.5	9532	0.0182	50
9.728	713.7	0.01363	60
24.58	1793	0.01371	60
39.43	2884	0.01367	60
54.27	3974	0.01366	60
69.11	5071	0.01363	60
83.95	6176	0.01359	60
98.79	7284	0.01356	60
113.6	8416	0.0135	60
128.5	9569	0.01342	60
143.3	10,710	0.01338	60
9.647	920.2	0.01048	70
24.37	2330	0.01046	70
39.1	3734	0.01047	70
53.82	5156	0.01044	70
68.53	6601	0.01038	70
83.24	8057	0.01033	70
97.94	9514	0.01029	70
112.6	10,970	0.01026	70

**Fig. 3:**
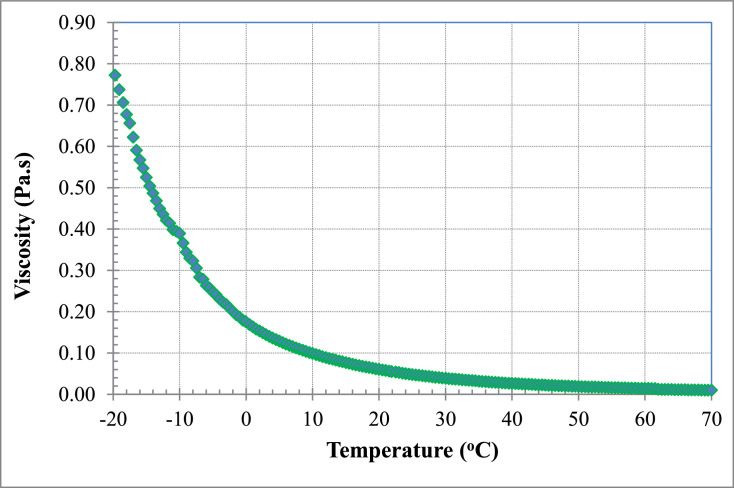
Viscosity versus temperature for pure PAO base fluid over a temperature range from negative 20 to 70 °C.

Tables 2, 4 and 6 (data set (2)) provide the raw data for pure PAO base fluid (*ϕ* = 0), for PAO/hBN nanofluids with BN particle concentration, *ϕ* = 0.6% and *ϕ* = 1.0%, respectively as a function of temperature at fixed shear stress and varied temperature at 0.5 °C intervals. This set of data is used to derive correlations for the viscosity as a function of temperature. Figs. 3, 5 and 7 illustrate the data of Tables 2, 4 and 6, respectively in terms of viscosity versus temperature for pure PAO base fluids (*ϕ* = 0%), for PAO/hBN nanofluids with BN particle concentration, *ϕ* = 0.6%, and 1.0%, respectively over the full temperature range from negative 20 to 70 °C.

**Table 2:** Raw data for pure PAO base fluids (*ϕ* = 0) as a function of temperature at fixed shear stress and varied temperature at 0.5 °C intervals. This set of data is used to derive correlations for the viscosity as a function of temperature.

Shear stress	Shear rate	Viscosity	Temperature
Pa	1/s	Pa.s	°C
9.995	12.94	0.77220	−19.70
9.995	13.55	0.73740	−19.10
9.995	14.15	0.70620	−18.50
9.994	14.75	0.67740	−18.00
9.994	15.23	0.65610	−17.50
9.994	16.07	0.62200	−17.00
9.994	16.92	0.59070	−16.50
9.993	17.62	0.56710	−16.00
9.993	18.26	0.54710	−15.50
9.993	19.03	0.52500	−15.00
9.992	19.83	0.50400	−14.50
9.992	20.55	0.48630	−14.00
9.992	21.35	0.46800	−13.50
9.991	22.24	0.44920	−13.00
9.991	22.93	0.43570	−12.50
9.991	23.74	0.42090	−12.00
9.991	24.16	0.41350	−11.50
9.990	25.09	0.39820	−11.00
9.990	25.35	0.39410	−10.50
9.990	25.67	0.38910	−10.00
9.990	27.29	0.36600	−9.50
9.989	29.07	0.34360	−9.00
9.988	30.32	0.32950	−8.50
9.988	30.95	0.32270	−8.00
9.987	32.70	0.30550	−7.50
9.987	35.25	0.28330	−7.00
9.986	35.90	0.27820	−6.50
9.985	37.94	0.26320	−6.00
9.985	38.82	0.25720	−5.50
9.985	40.42	0.24700	−5.00
9.984	41.40	0.24120	−4.50
9.983	43.37	0.23020	−4.00
9.983	44.75	0.22310	−3.50
9.982	46.05	0.21680	−3.00
9.982	47.88	0.20850	−2.50
9.981	49.81	0.20040	−2.00
9.980	51.87	0.19240	−1.50
9.980	53.52	0.18650	−1.00
9.979	55.67	0.17920	−0.50
9.978	56.66	0.17610	−0.10
9.977	59.21	0.16850	0.50
9.977	61.07	0.16340	1.00
9.976	63.26	0.15770	1.50
9.975	65.18	0.15300	2.00
9.974	67.16	0.14850	2.50
9.974	69.21	0.14410	3.00
9.973	71.23	0.14000	3.50
9.972	73.28	0.13610	4.00
9.971	75.34	0.13230	4.50
9.970	77.55	0.12860	5.00
9.969	79.79	0.12490	5.50
9.969	81.91	0.12170	6.00
9.968	84.28	0.11830	6.50
9.967	86.53	0.11520	7.00
9.966	88.83	0.11220	7.50
9.965	91.33	0.10910	8.00
9.964	93.74	0.10630	8.50
9.963	96.20	0.10360	9.00
9.962	98.63	0.10100	9.50
9.961	101.20	0.09844	10.00
9.960	103.90	0.09588	10.50
9.959	106.50	0.09354	11.00
9.958	109.30	0.09113	11.50
9.957	112.00	0.08889	12.00
9.956	114.90	0.08668	12.50
9.955	117.80	0.08454	13.00
9.954	120.80	0.08242	13.50
9.953	123.70	0.08046	14.00
9.951	126.80	0.07849	14.50
9.950	129.90	0.07662	15.00
9.949	133.30	0.07466	15.50
9.948	136.60	0.07283	16.00
9.946	139.90	0.07110	16.50
9.945	143.50	0.06931	17.00
9.944	147.00	0.06767	17.50
9.942	150.50	0.06605	18.00
9.941	154.10	0.06453	18.50
9.940	157.80	0.06301	19.00
9.938	161.50	0.06153	19.50
9.937	165.30	0.06011	20.00
9.935	169.20	0.05871	20.50
9.934	173.30	0.05732	21.00
9.932	177.30	0.05602	21.50
9.931	181.20	0.05481	21.90
9.929	185.60	0.05349	22.50
9.927	189.70	0.05234	23.00
9.926	194.00	0.05116	23.50
9.924	198.60	0.04997	24.00
9.922	203.10	0.04886	24.50
9.921	207.70	0.04775	25.00
9.919	212.50	0.04669	25.50
9.917	216.80	0.04575	26.00
9.915	221.40	0.04478	26.50
9.913	226.20	0.04383	27.00
9.912	231.10	0.04290	27.50
9.910	236.10	0.04197	28.00
9.908	241.00	0.04111	28.50
9.906	246.30	0.04023	29.00
9.904	251.60	0.03936	29.50
9.902	256.70	0.03858	30.00
9.900	262.00	0.03778	30.50
9.898	266.90	0.03709	31.00
9.896	272.10	0.03637	31.50
9.894	277.20	0.03569	32.00
9.892	282.90	0.03496	32.50
9.890	288.50	0.03428	33.00
9.888	294.00	0.03363	33.50
9.885	300.00	0.03295	34.00
9.883	305.80	0.03232	34.50
9.881	311.90	0.03168	35.00
9.878	317.90	0.03108	35.50
9.876	323.80	0.03050	36.00
9.874	330.00	0.02992	36.50
9.871	336.20	0.02936	37.00
9.869	342.50	0.02881	37.50
9.866	348.90	0.02828	38.00
9.864	355.30	0.02776	38.50
9.862	362.00	0.02724	39.00
9.859	368.70	0.02674	39.50
9.856	375.10	0.02628	40.00
9.854	382.40	0.02577	40.50
9.851	388.50	0.02535	41.00
9.849	395.80	0.02488	41.50
9.846	402.90	0.02444	42.00
9.843	409.80	0.02402	42.50
9.840	417.30	0.02358	43.00
9.838	424.30	0.02318	43.50
9.835	431.50	0.02279	44.00
9.832	438.80	0.02240	44.50
9.829	446.40	0.02202	45.00
9.826	454.00	0.02164	45.50
9.824	461.10	0.02130	46.00
9.821	468.80	0.02095	46.50
9.817	477.20	0.02057	47.00
9.815	484.70	0.02025	47.50
9.812	492.50	0.01992	48.00
9.808	500.60	0.01959	48.50
9.805	508.80	0.01927	49.00
9.802	516.70	0.01897	49.50
9.799	524.70	0.01868	50.00
9.796	533.00	0.01838	50.50
9.793	540.70	0.01811	51.00
9.790	549.40	0.01782	51.50
9.787	557.60	0.01755	52.00
9.784	564.60	0.01733	52.50
9.780	573.80	0.01704	53.00
9.777	581.70	0.01681	53.50
9.774	590.60	0.01655	54.00
9.771	598.60	0.01632	54.50
9.768	606.60	0.01610	55.00
9.765	615.00	0.01588	55.50
9.761	623.80	0.01565	56.00
9.758	631.30	0.01546	56.50
9.755	641.40	0.01521	57.00
9.752	649.00	0.01502	57.50
9.749	657.00	0.01484	58.00
9.746	663.90	0.01468	58.50
9.743	670.90	0.01452	59.00
9.739	681.90	0.01428	59.50
9.736	690.50	0.01410	60.00
9.734	695.10	0.01400	60.50
9.733	698.80	0.01393	61.00
9.729	709.30	0.01372	61.50
9.687	817.10	0.01186	62.00
9.683	827.50	0.01170	62.50
9.680	837.40	0.01156	63.00
9.675	848.60	0.01140	63.50
9.671	858.80	0.01126	64.00
9.667	869.10	0.01112	64.50
9.663	880.70	0.01097	65.00
9.659	891.60	0.01083	65.50
9.655	902.20	0.01070	66.00
9.650	913.60	0.01056	66.50
9.646	924.70	0.01043	67.00
9.642	935.60	0.01031	67.50
9.637	948.00	0.01017	68.00
9.633	958.80	0.01005	68.50
9.628	971.30	0.00991	69.00
9.624	982.70	0.00979	69.50
9.620	994.50	0.00967	70.00

Fig. 2 shows a plot of Table 1 data of the sear stress versus shear rate for the base fluid (pure PAO) over the full range of temperatures from negative 20 to 70 °C. The linear behaviour of the results confirms that the pure PAO is Newtonian fluid.

Fig. 3 illustrates data from Table 2 of the dependence of viscosity on temperature for the base fluid (pure PAO) over a temperature range from negative 20 to 70 °C.

**Fig. 4:**
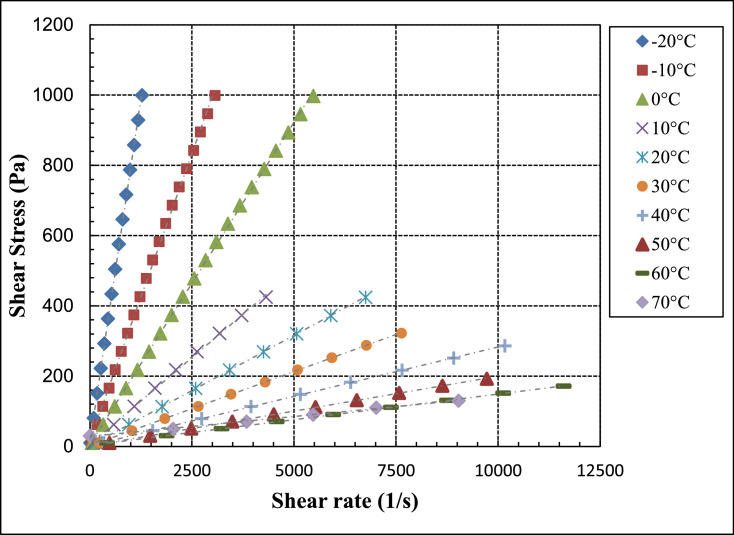
Shear stress versus shear strain for PAO/hBN nanofluid with BN particle concentration, *ϕ* = 0.6% over a temperature range from negative 20 to 70 °C.

Fig. 4 shows a plot of the data presented in Table 3 of the sear stress versus shear rate for PAO/hBN nanofluid with BN particle concentration, *ϕ* = 0.6% over the full range of temperatures from negative 20 to 70 °C.

**Table 3:** Raw data for PAO/hBN nanofluid for BN particle concentration, *ϕ* = 0.6% as a function of temperature at fixed temperature intervals and varied shear and shear rate. This set of data is used to verify whether the fluid is Newtonian or non-Newtonian.

Shear stress	Shear rate	Viscosity	Temperature
Pa	1/s	Pa.s	°C
9.996	11.74	0.8513	−20
80.68	95.4	0.8457	−20
151.4	180.6	0.8379	−20
222	267.1	0.8314	−20
292.7	354.7	0.8253	−20
363.4	445.1	0.8164	−20
434.1	534	0.8128	−20
504.8	622.2	0.8113	−20
575.4	711.3	0.809	−20
646.1	801.3	0.8064	−20
716.8	893.9	0.8019	−20
787.5	988.1	0.7969	−20
858.2	1087	0.7893	−20
928.8	1182	0.7856	−20
999.5	1278	0.7823	−20
9.989	27.57	0.3623	−10
62.04	172.5	0.3597	−10
114.1	319.6	0.3569	−10
166.1	470.2	0.3533	−10
218.2	618.6	0.3527	−10
270.2	771.9	0.3501	−10
322.3	924.7	0.3485	−10
374.3	1077	0.3474	−10
426.4	1229	0.3469	−10
478.4	1385	0.3455	−10
530.5	1539	0.3446	−10
582.5	1700	0.3426	−10
634.6	1859	0.3413	−10
686.6	2016	0.3405	−10
738.6	2192	0.337	−10
790.7	2364	0.3344	−10
842.7	2541	0.3316	−10
894.8	2710	0.3302	−10
946.8	2885	0.3282	−10
998.8	3068	0.3256	−10
9.98	51.63	0.1933	0
61.98	328.8	0.1885	0
114	609.9	0.1869	3.00E-03
166	888.9	0.1867	3.00E-03
218	1168	0.1867	7.00E-03
270	1448	0.1864	3.00E-03
322	1726	0.1866	3.00E-03
374	2000	0.187	7.00E-03
426	2279	0.1869	−2.00E-03
478	2558	0.1868	0
530	2836	0.1869	−0.01
582	3100	0.1878	−6.00E-03
634	3384	0.1874	−2.00E-03
686	3674	0.1867	0
738	3971	0.1859	3.00E-03
789.9	4270	0.185	−2.00E-03
841.9	4559	0.1847	7.00E-03
893.9	4861	0.1839	−6.00E-03
945.9	5161	0.1833	−0.01
997.9	5475	0.1823	0
9.964	93.13	0.107	10
61.88	589.6	0.1049	10
113.8	1089	0.1045	10
165.7	1595	0.1039	10
217.6	2107	0.1033	10
269.5	2628	0.1025	10
321.4	3178	0.1011	10
373.3	3721	0.1003	10
425.2	4314	0.09856	10
9.941	154.2	0.06447	20
61.74	959.1	0.06437	20
113.5	1770	0.06414	20
165.3	2591	0.06381	20
217.1	3427	0.06336	20
268.9	4253	0.06323	20
320.7	5069	0.06326	20
372.5	5901	0.06312	20
424.3	6760	0.06276	20
9.912	230	0.0431	30
44.61	1032	0.04323	30
79.3	1836	0.0432	30
114	2660	0.04286	30
148.7	3459	0.04298	30
183.4	4296	0.04268	30
218	5092	0.04282	30
252.7	5929	0.04263	30
287.4	6772	0.04244	30
322.1	7636	0.04218	30
9.87	341.5	0.0289	40
44.41	1539	0.02886	40
78.95	2742	0.0288	40
113.5	3947	0.02876	40
148	5161	0.02868	40
182.6	6390	0.02857	40
217.1	7649	0.02838	40
251.6	8915	0.02822	40
286.1	10,170	0.02813	40
9.817	478.4	0.02052	50
30.15	1482	0.02034	50
50.48	2485	0.02031	50
70.81	3492	0.02027	50
91.14	4509	0.02021	50
111.5	5530	0.02016	50
131.8	6537	0.02016	50
152.1	7579	0.02007	50
172.4	8636	0.01996	50
192.7	9725	0.01982	50
9.832	427.6	0.02299	60
29.99	1881	0.01595	60
50.19	3233	0.01552	60
70.39	4578	0.01538	60
90.58	5964	0.01519	60
110.7	7378	0.01501	60
130.9	8752	0.01496	60
151.1	10,130	0.01492	60
171.3	11,610	0.01476	60
10	5.06E-06	1.98E+06	70
29.93	2041	0.01467	70
49.96	3841	0.01301	70
70.05	5469	0.01281	70
90.18	7016	0.01285	70
110.1	9034	0.01219	70
130.1	10,970	0.01186	70

**Fig. 5:**
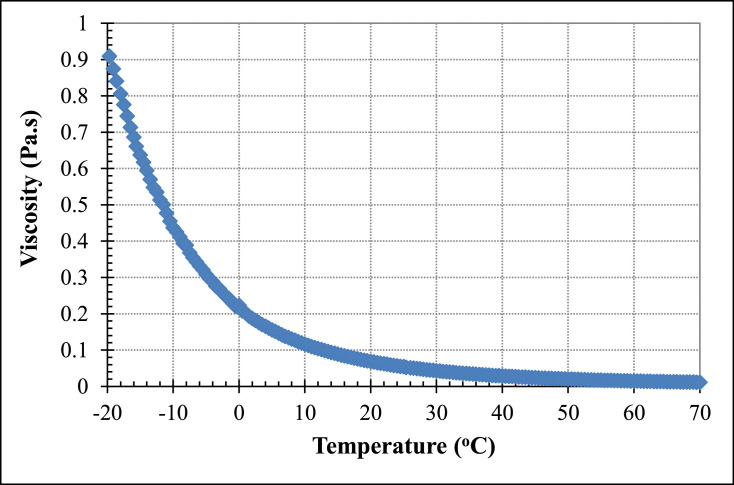
Viscosity versus temperature for PAO/hBN nanofluid with BN particle concentration, *ϕ* = 0.6% over a temperature range from negative 20 to 70 °C.

Fig. 5 illustrates data from Table 4 of the dependence of viscosity on temperature for PAO/hBN nanofluid with BN particle concentration, *ϕ* = 0.6% over a temperature range from negative 20 to 70 °C.

**Table 4:** Raw data for PAO/hBN nanofluid with BN particle concentration, *ϕ* = 0.6% as a function of temperature at fixed shear stress and varied temperature at 0.5 °C intervals. This set of data is used to derive correlations for the viscosity as a function of temperature.

Shear stress	Shear rate	Viscosity	Temperature
Pa	1/s	Pa.s	°C
9.996	10.99	0.90920	−19.70
9.996	11.43	0.87420	−19.10
9.995	11.89	0.84070	−18.60
9.995	12.40	0.80590	−18.00
9.995	12.88	0.77630	−17.50
9.995	13.42	0.74460	−17.00
9.995	14.01	0.71340	−16.50
9.994	14.56	0.68640	−16.00
9.994	15.13	0.66060	−15.60
9.994	15.68	0.63730	−15.00
9.994	16.20	0.61680	−14.50
9.994	16.82	0.59410	−14.00
9.993	17.54	0.56980	−13.50
9.993	18.24	0.54780	−13.00
9.993	18.69	0.53470	−12.50
9.993	19.45	0.51360	−12.00
9.992	19.95	0.50090	−11.50
9.992	20.95	0.47690	−11.00
9.992	21.97	0.45470	−10.50
9.991	22.87	0.43690	−10.00
9.991	23.48	0.42550	−9.50
9.991	24.25	0.41200	−9.00
9.990	25.37	0.39380	−8.50
9.990	25.70	0.38880	−8.00
9.990	27.15	0.36800	−7.50
9.989	28.16	0.35470	−7.00
9.989	29.09	0.34340	−6.50
9.988	30.10	0.33190	−6.00
9.988	31.14	0.32080	−5.50
9.988	32.42	0.30810	−5.00
9.987	33.63	0.29700	−4.50
9.987	34.62	0.28840	−4.00
9.986	36.19	0.27590	−3.50
9.986	37.07	0.26930	−3.00
9.985	38.70	0.25800	−2.50
9.985	40.25	0.24810	−2.00
9.984	41.40	0.24120	−1.50
9.983	43.54	0.22930	−1.00
9.983	45.30	0.22030	−0.50
9.983	44.76	0.22300	0.00
9.982	48.01	0.20790	0.50
9.981	49.51	0.20160	1.00
9.980	51.50	0.19380	1.50
9.980	53.33	0.18710	2.00
9.979	55.01	0.18140	2.50
9.978	56.81	0.17560	3.00
9.978	58.64	0.17010	3.50
9.977	60.41	0.16520	4.00
9.976	62.21	0.16040	4.50
9.975	64.28	0.15520	5.00
9.975	66.07	0.15100	5.50
9.974	68.02	0.14660	6.00
9.973	70.19	0.14210	6.50
9.972	72.28	0.13800	7.00
9.972	74.21	0.13440	7.50
9.971	76.54	0.13030	8.00
9.970	78.62	0.12680	8.50
9.969	81.08	0.12300	9.00
9.968	83.44	0.11950	9.50
9.967	85.84	0.11610	10.00
9.966	88.41	0.11270	10.50
9.965	90.72	0.10980	11.00
9.964	93.21	0.10690	11.50
9.963	95.74	0.10410	12.00
9.962	98.32	0.10130	12.50
9.961	101.00	0.09858	13.00
9.960	103.70	0.09602	13.50
9.959	106.60	0.09338	14.00
9.958	109.40	0.09102	14.50
9.957	112.40	0.08862	15.00
9.956	115.50	0.08619	15.50
9.955	118.30	0.08412	16.00
9.953	121.70	0.08182	16.50
9.952	124.50	0.07994	17.00
9.951	128.00	0.07773	17.50
9.950	131.00	0.07596	18.00
9.948	134.80	0.07380	18.50
9.947	138.00	0.07209	19.00
9.946	141.30	0.07040	19.50
9.944	145.20	0.06850	20.00
9.943	148.20	0.06708	20.50
9.942	152.30	0.06529	21.00
9.940	155.80	0.06381	21.50
9.939	159.50	0.06231	22.00
9.937	163.80	0.06069	22.50
9.936	167.70	0.05924	23.00
9.934	171.60	0.05789	23.50
9.933	175.90	0.05646	24.00
9.931	179.70	0.05525	24.50
9.930	183.90	0.05400	25.00
9.927	190.60	0.05207	25.40
9.926	194.60	0.05101	26.00
9.924	198.40	0.05002	26.50
9.922	202.70	0.04895	27.00
9.921	207.20	0.04789	27.50
9.919	211.80	0.04682	28.00
9.917	216.80	0.04573	28.50
9.915	221.20	0.04482	29.00
9.914	225.80	0.04390	29.50
9.912	231.20	0.04286	30.00
9.910	235.60	0.04206	30.50
9.908	241.00	0.04111	31.00
9.906	246.30	0.04021	31.50
9.904	251.30	0.03941	32.00
9.902	256.80	0.03856	32.50
9.900	262.30	0.03774	33.00
9.898	267.70	0.03697	33.50
9.896	272.70	0.03629	34.00
9.893	278.80	0.03548	34.50
9.891	284.60	0.03476	35.00
9.889	290.00	0.03411	35.50
9.887	296.40	0.03335	36.00
9.884	302.10	0.03272	36.50
9.882	307.70	0.03211	37.00
9.880	313.60	0.03150	37.50
9.878	320.10	0.03086	38.00
9.875	326.00	0.03029	38.50
9.873	332.50	0.02969	39.00
9.870	338.90	0.02913	39.50
9.868	345.20	0.02858	40.00
9.866	351.40	0.02807	40.50
9.863	358.40	0.02752	41.00
9.860	365.00	0.02702	41.50
9.858	371.90	0.02650	42.00
9.855	378.80	0.02602	42.50
9.853	385.10	0.02559	43.00
9.850	392.40	0.02510	43.50
9.847	399.70	0.02464	44.00
9.844	406.50	0.02422	44.50
9.842	413.80	0.02378	45.00
9.839	421.30	0.02336	45.50
9.836	429.00	0.02293	46.00
9.833	435.90	0.02256	46.50
9.830	443.20	0.02218	47.00
9.828	450.90	0.02180	47.50
9.824	459.10	0.02140	48.00
9.822	466.10	0.02107	48.50
9.819	474.40	0.02070	49.00
9.816	482.00	0.02037	49.50
9.813	489.70	0.02004	50.00
9.809	498.30	0.01968	50.50
9.806	506.00	0.01938	51.00
9.803	514.20	0.01907	51.50
9.800	522.70	0.01875	52.00
9.797	530.60	0.01846	52.50
9.794	539.40	0.01816	53.00
9.790	548.00	0.01787	53.50
9.787	556.40	0.01759	54.00
9.784	565.10	0.01731	54.50
9.780	573.80	0.01705	55.00
9.777	582.40	0.01679	55.50
9.774	591.50	0.01652	56.00
9.770	600.40	0.01627	56.50
9.767	609.00	0.01604	57.00
9.764	617.90	0.01580	57.50
9.760	627.80	0.01555	58.00
9.757	636.40	0.01533	58.50
9.753	646.30	0.01509	59.00
9.749	655.20	0.01488	59.50
9.746	664.60	0.01466	60.00
9.742	674.20	0.01445	60.50
9.738	684.00	0.01424	61.00
9.735	693.80	0.01403	61.50
9.731	702.30	0.01386	62.00
9.727	712.50	0.01365	62.50
9.723	723.20	0.01345	63.00
9.720	732.30	0.01327	63.50
9.716	741.60	0.01310	64.00
9.712	752.30	0.01291	64.50
9.709	760.20	0.01277	65.00
9.705	770.40	0.01260	65.50
9.702	779.70	0.01244	66.00
9.698	789.50	0.01228	66.50
9.694	799.40	0.01213	67.00
9.691	808.70	0.01198	67.50
9.686	820.00	0.01181	68.00
9.683	828.80	0.01168	68.50
9.679	838.00	0.01155	69.00
9.675	849.80	0.01139	69.50
9.672	857.50	0.01128	70.00

**Fig. 6:**
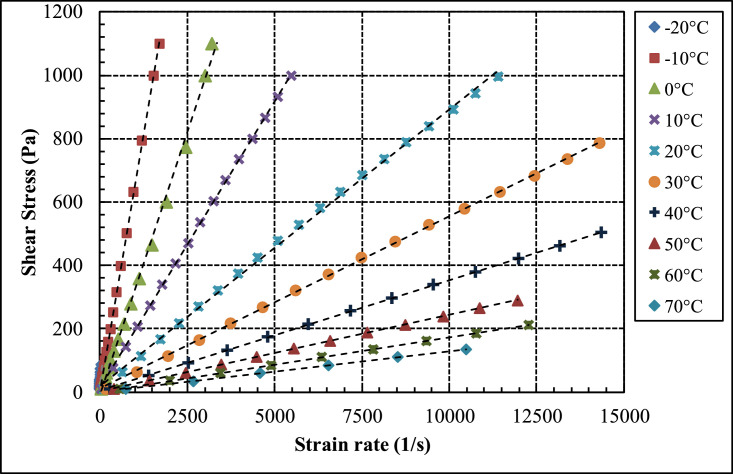
Shear stress versus shear strain for PAO/hBN nanofluid with BN particle concentration, *ϕ* = 1.0% over a temperature range from negative 20 to 70 °C. This figure is duplicated from research article published by the author in [1].

Fig. 6 shows a plot of the data presented in Table 5 of the sear stress versus shear rate for PAO/hBN nanofluid with BN particle concentration, *ϕ* = 1.0% over the full range of temperatures from negative 20 to 70 °C.

**Table 5:** Raw data for PAO/hBN nanofluid for BN particle concentration, *ϕ* = 1.0% as a function of temperature at fixed temperature intervals and varied shear and shear rate. This set of data is used to verify whether the fluid is Newtonian or non-Newtonian.

Shear stress	Shear rate	Viscosity	Temperature
Pa	1/s	Pa.s	°C
9.998	5.864	1.705	−20
11.66	6.873	1.696	−20
13.59	8.021	1.694	−20
15.85	9.343	1.696	−20
18.47	10.92	1.692	−20
21.54	12.71	1.694	−20
25.11	14.91	1.684	−20
29.28	17.41	1.682	−20
34.14	20.27	1.684	−20
39.8	23.65	1.683	−20
46.41	27.63	1.679	−20
54.1	32.36	1.672	−20
63.08	37.72	1.672	−20
73.55	44.18	1.665	−20
85.75	51.5	1.665	−20
9.995	14.09	0.7096	−10
12.58	17.79	0.7072	−10
15.84	22.46	0.7053	−10
19.94	28.31	0.7044	−10
25.11	35.62	0.7047	−10
31.61	45.07	0.7012	−10
39.79	57.09	0.6969	−10
50.09	72.18	0.694	−10
63.06	91.57	0.6887	−10
79.39	115.7	0.686	−10
99.94	146	0.6843	−10
125.8	184.3	0.6827	−10
158.4	232.6	0.681	−10
199.4	295.8	0.6741	−10
251	372.9	0.6731	−10
316	471.8	0.6699	−10
397.9	597.8	0.6656	−10
500.9	755.4	0.6631	−10
630.6	950.1	0.6637	−10
793.9	1208	0.657	−10
999.4	1541	0.6485	−10
1099	1705	0.6447	−10
9.988	31.07	0.3215	7.00E-03
12.9	40.3	0.3201	−2.00E-03
16.66	52.14	0.3195	−2.00E-03
21.52	67.52	0.3187	−2.00E-03
27.79	87.8	0.3165	0
35.89	113.6	0.3158	0
46.36	147.4	0.3145	−6.00E-03
59.88	190.6	0.3142	3.00E-03
77.33	243.9	0.3171	7.00E-03
99.88	314.2	0.3179	−0.01
129	406.3	0.3175	−2.00E-03
166.6	525.1	0.3173	3.00E-03
215.2	680.1	0.3164	−6.00E-03
277.9	876.9	0.3169	3.00E-03
358.9	1130	0.3175	−6.00E-03
463.6	1476	0.3142	7.00E-03
598.8	1895	0.316	3.00E-03
773.3	2456	0.3148	0
998.9	3017	0.3311	0
1099	3203	0.343	−2.00E-03
9.981	49.21	0.2028	10
75.85	385.8	0.1966	10
141.7	727.5	0.1948	10
207.6	1077	0.1928	10
273.5	1434	0.1907	10
339.3	1790	0.1896	10
405.2	2157	0.1879	10
471	2519	0.187	10
536.9	2874	0.1868	10
602.8	3241	0.186	10
668.6	3599	0.1858	10
734.5	3973	0.1849	10
800.3	4363	0.1834	10
866.2	4724	0.1834	10
932.1	5098	0.1828	10
997.9	5471	0.1824	10
9.961	103.5	0.09625	20
61.86	639.9	0.09668	20
113.8	1178	0.09655	20
165.7	1724	0.09609	20
217.6	2267	0.09595	20
269.4	2828	0.09527	20
321.3	3385	0.09493	20
373.2	3945	0.0946	20
425.1	4521	0.09404	20
477	5085	0.09381	20
528.9	5689	0.09297	20
580.8	6292	0.0923	20
632.6	6883	0.09191	20
684.5	7503	0.09123	20
736.4	8132	0.09055	20
788.2	8776	0.08981	20
840.1	9412	0.08926	20
891.9	10,110	0.08822	20
943.8	10,750	0.08781	20
995.6	11,400	0.08733	20
9.936	168.6	0.05893	30
61.7	1048	0.0589	30
113.5	1938	0.05854	30
165.2	2831	0.05836	30
217	3735	0.05809	30
268.7	4661	0.05766	30
320.5	5593	0.05731	30
372.2	6553	0.05681	30
424	7484	0.05665	30
475.7	8444	0.05634	30
527.4	9424	0.05597	30
579.2	10,440	0.0555	30
630.9	11,440	0.05517	30
682.6	12,430	0.05492	30
734.4	13,380	0.05488	30
786.1	14,300	0.05497	30
837.9	15,270	0.05488	30
889.6	16,070	0.05535	30
9.896	271.2	0.03649	40
51.05	1396	0.03657	40
92.19	2528	0.03647	40
133.3	3648	0.03655	40
174.5	4809	0.03628	40
215.6	5972	0.0361	40
256.7	7172	0.0358	40
297.9	8346	0.03569	40
339	9528	0.03558	40
380.1	10,760	0.03532	40
421.2	11,980	0.03516	40
462.3	13,170	0.03511	40
503.4	14,350	0.03507	40
9.848	396.4	0.02484	50
35.25	1420	0.02482	50
60.64	2443	0.02482	50
86.04	3471	0.02479	50
111.4	4495	0.02479	50
136.8	5551	0.02465	50
162.2	6598	0.02458	50
187.6	7664	0.02448	50
213	8733	0.02439	50
238.4	9824	0.02426	50
263.7	10,880	0.02423	50
289.1	11,950	0.02419	50
9.785	560	0.01747	60
35.02	2002	0.01749	60
60.26	3447	0.01748	60
85.5	4894	0.01747	60
110.7	6344	0.01745	60
136	7830	0.01736	60
161.2	9339	0.01726	60
186.4	10,780	0.01728	60
211.6	12,280	0.01724	60
9.714	747.5	0.01299	70
34.77	2663	0.01306	70
59.82	4584	0.01305	70
84.87	6548	0.01296	70
109.9	8525	0.01289	70
134.9	10,480	0.01288	70

**Fig. 7:**
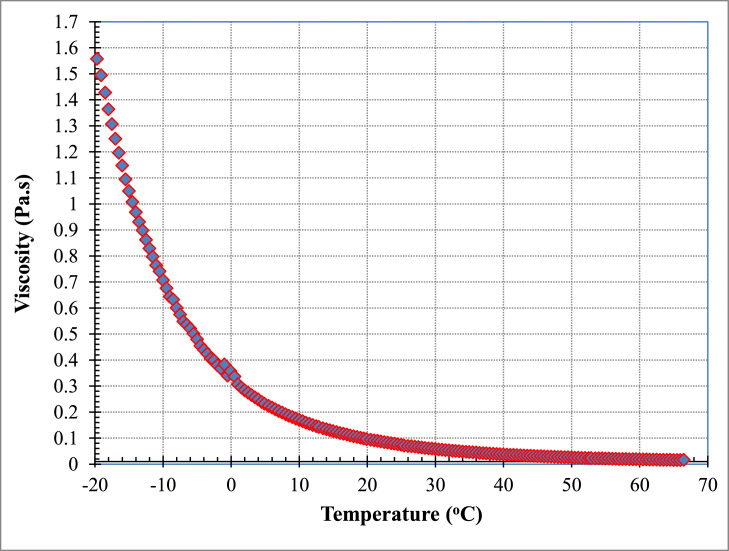
Viscosity versus temperature for PAO/hBN nanofluid with BN particle concentration, *ϕ* = 1.0% over a temperature range from negative 20 to 68.5 °C.

Fig. 7 illustrates data from Table 6 of the dependence of viscosity on temperature for PAO/hBN nanofluid with BN particle concentration, *ϕ* = 1.0% over a temperature range from negative 20 to 68.5 °C.

**Table 6:** Raw data for PAO/hBN nanofluid with BN particle concentration, *ϕ* = 1.0% as a function of temperature at fixed shear stress and varied temperature at 0.5 °C intervals. This set of data is used to derive correlations for the viscosity as a function of temperature.

Shear stress	Shear rate	Viscosity	Temperature
Pa	1/s	Pa.s	°C
9.998	6.42	1.55700	−19.70
9.997	6.69	1.49400	−19.10
9.997	7.00	1.42800	−18.50
9.997	7.33	1.36400	−18.00
9.997	7.65	1.30700	−17.50
9.997	8.00	1.25000	−17.00
9.997	8.36	1.19600	−16.50
9.997	8.72	1.14700	−16.00
9.997	9.14	1.09400	−15.50
9.996	9.53	1.04900	−15.00
9.996	9.93	1.00700	−14.50
9.996	10.33	0.96800	−14.00
9.996	10.74	0.93080	−13.50
9.996	11.13	0.89820	−13.00
9.996	11.59	0.86220	−12.50
9.995	12.06	0.82890	−12.00
9.995	12.53	0.79740	−11.50
9.995	13.08	0.76420	−11.00
9.995	13.50	0.74050	−10.50
9.995	14.14	0.70690	−10.00
9.994	14.78	0.67640	−9.50
9.994	15.54	0.64330	−9.00
9.994	15.81	0.63200	−8.50
9.994	16.64	0.60050	−8.00
9.993	17.38	0.57510	−7.50
9.993	18.23	0.54820	−7.00
9.993	18.65	0.53580	−6.50
9.993	19.18	0.52090	−6.00
9.992	19.94	0.50100	−5.50
9.992	20.84	0.47950	−5.00
9.992	21.96	0.45490	−4.50
9.991	22.71	0.43990	−4.00
9.991	23.55	0.42420	−3.50
9.991	24.63	0.40570	−3.00
9.990	25.06	0.39860	−2.50
9.990	26.29	0.38000	−2.00
9.990	27.22	0.36700	−1.50
9.990	26.10	0.38280	−1.00
9.989	29.34	0.34040	−0.50
9.989	27.97	0.35710	−0.10
9.989	29.70	0.33630	0.50
9.988	32.52	0.30710	1.00
9.987	33.89	0.29470	1.50
9.987	35.22	0.28360	2.00
9.986	36.37	0.27460	2.50
9.986	37.62	0.26540	3.00
9.985	38.92	0.25660	3.50
9.985	40.15	0.24870	4.00
9.984	41.76	0.23910	4.50
9.984	43.07	0.23180	4.90
9.983	44.54	0.22410	5.50
9.982	45.92	0.21740	6.00
9.982	47.36	0.21080	6.50
9.981	48.86	0.20430	7.00
9.981	50.47	0.19780	7.50
9.980	52.08	0.19160	8.00
9.979	53.72	0.18580	8.50
9.979	55.34	0.18030	9.00
9.978	57.07	0.17490	9.50
9.977	58.86	0.16950	10.00
9.977	60.63	0.16460	10.50
9.976	62.56	0.15950	11.00
9.975	64.36	0.15500	11.50
9.975	66.31	0.15040	12.00
9.974	68.25	0.14610	12.50
9.973	70.13	0.14220	12.90
9.972	72.30	0.13790	13.50
9.972	74.41	0.13400	14.00
9.971	76.49	0.13030	14.50
9.970	78.77	0.12660	15.00
9.969	80.91	0.12320	15.50
9.968	83.29	0.11970	16.00
9.967	85.64	0.11640	16.50
9.966	87.99	0.11330	17.00
9.965	90.72	0.10990	17.50
9.964	93.07	0.10710	18.00
9.963	95.49	0.10430	18.50
9.962	98.29	0.10140	19.00
9.961	100.80	0.09880	19.50
9.960	103.30	0.09639	19.90
9.959	106.30	0.09370	20.50
9.958	109.00	0.09138	21.00
9.957	111.80	0.08907	21.50
9.956	114.80	0.08669	22.00
9.955	117.90	0.08444	22.50
9.954	120.80	0.08240	23.00
9.953	123.90	0.08033	23.50
9.951	127.00	0.07834	24.00
9.950	130.30	0.07636	24.50
9.949	133.60	0.07445	25.00
9.947	139.40	0.07137	25.40
9.945	142.50	0.06978	26.00
9.944	145.60	0.06830	26.50
9.943	149.20	0.06664	27.00
9.942	152.80	0.06507	27.50
9.940	156.40	0.06358	28.00
9.939	160.00	0.06211	28.50
9.937	163.80	0.06065	29.00
9.936	167.60	0.05927	29.50
9.934	171.90	0.05780	30.00
9.933	175.50	0.05661	30.50
9.931	179.50	0.05532	31.00
9.930	184.00	0.05396	31.50
9.928	188.30	0.05272	32.00
9.926	192.30	0.05162	32.50
9.925	196.90	0.05040	33.00
9.923	201.40	0.04926	33.50
9.921	205.80	0.04820	34.00
9.920	210.20	0.04720	34.50
9.918	214.70	0.04619	35.00
9.916	219.50	0.04517	35.50
9.914	224.20	0.04422	36.00
9.912	229.10	0.04327	36.50
9.911	233.90	0.04237	37.00
9.909	238.80	0.04149	37.50
9.907	244.10	0.04058	38.00
9.905	249.00	0.03977	38.50
9.903	254.00	0.03899	39.00
9.901	259.70	0.03812	39.50
9.899	264.60	0.03741	40.00
9.897	270.30	0.03661	40.50
9.894	275.90	0.03586	41.00
9.892	281.00	0.03520	41.50
9.890	287.20	0.03444	42.00
9.888	292.40	0.03382	42.50
9.886	298.30	0.03314	43.00
9.884	304.10	0.03250	43.50
9.881	310.40	0.03184	44.00
9.879	315.80	0.03128	44.50
9.877	321.60	0.03071	45.00
9.875	328.00	0.03011	45.50
9.872	333.90	0.02956	46.00
9.870	340.30	0.02900	46.50
9.867	346.70	0.02846	47.00
9.865	352.90	0.02796	47.50
9.862	359.50	0.02743	48.00
9.860	366.10	0.02694	48.50
9.857	372.60	0.02645	49.00
9.855	379.50	0.02597	49.50
9.852	386.50	0.02549	50.00
9.849	393.40	0.02504	50.50
9.847	399.90	0.02462	51.00
9.844	407.00	0.02419	51.50
9.842	414.20	0.02376	52.00
9.839	421.50	0.02334	52.50
9.836	428.10	0.02298	52.90
9.833	435.50	0.02258	53.50
9.830	443.10	0.02218	54.00
9.828	450.10	0.02184	54.50
9.825	458.00	0.02145	55.00
9.822	464.90	0.02113	55.50
9.819	472.80	0.02077	56.00
9.816	480.10	0.02045	56.50
9.813	488.10	0.02010	57.00
9.810	496.50	0.01976	57.50
9.807	503.80	0.01947	58.00
9.804	511.50	0.01917	58.50
9.801	520.00	0.01885	59.00
9.798	527.80	0.01856	59.50
9.795	535.90	0.01828	60.00
9.792	543.60	0.01801	60.50
9.789	552.20	0.01773	61.00
9.786	560.10	0.01747	61.50
9.782	568.90	0.01720	62.00
9.779	577.40	0.01694	62.50
9.776	585.50	0.01670	63.00
9.773	594.00	0.01645	63.50
9.770	600.80	0.01626	64.00
9.767	607.90	0.01607	64.50
9.765	614.10	0.01590	65.00
9.764	617.00	0.01582	65.50
9.763	619.50	0.01576	66.00
9.767	609.90	0.01571	66.50
9.775	587.00	0.01566	67.00
9.807	504.90	0.01561	67.50
9.852	385.80	0.01556	68.00
9.851	212.00	0.01551	68.50

## 2 Experimental Design, Materials and Methods

The BN used in this study is the Hexagonal Boron Nitride (hBN) Powder 99.5% pure purchased from M K Impex Canada and the PAO is DURASYN_ 166 purchased from Chemcentral (Chicago, IL, USA). The average particles size of the BN is 70 nm and the density of the BN powder is 2.26 gm/cm3. Nanofluid samples with BN particle volumetric concentrations of 0.6% and 1% were prepared by adding the exact amount of Boron nitride to the PAO. For preparing the 1% by volume sample, a surfactant (oleic acid) was added in the amount of 50% by volume of BN particles. After that, the sample was placed on a magnetic stirrer for more than 30 min and then in ultrasonic agitator (Branson Digital Sonifier, model 450) for 360 min for the 0.6% samples and for 420 min for the 1% samples to ensure uniform dispersion of the nanoparticles.

Fig. 1 shows schematic of the experimental setup that was used to measure the rheological property of the pure PAO and the PAO/hBN nanofluids. The setup is an AR-G2 rheometer from TA Instruments, New Castle, Delaware. It is a combined motor and transducer (CMT) instrument. The lower component of the measuring system is fixed, while the upper component is attached to a shaft, that can be rotated by a torque produced by an induction motor. The constraint on the low torque performance of the instrument is the friction between the rotating and the stationary components. Standard 1° cone plate was used to measure the viscosity, shear stress and shear strain rate for the samples of the fluids. Measurements can be made at torques from 0.01 N.m to 200 mN-m. The fluid samples with 1.5 ml volume, is placed on the bottom fixed plate, then the upper movable part, which consists of cone assembly is moved to bottom. The distance between the two plates is about 26 µ.m. The desired shear stress range is produced by moving the cone plate over the fluid. To control the temperature of the test fluid fromn −20 °C to 70 °C, a water circulator chamber is used. The measure data include rotational speed of the spindle (RPM), torque, viscosity (Pa.s), shear stress (Pa), shear strain rate (1/s) and temperature (°C).

Each viscosity measurement was conducted under thermal equilibrium by insuring that sufficient time (at least 3 min) is given between measurements to allow the temperature to stabilize.

## Ethics Statement

The work did not involve the use of human subjects nor animal experiments. The work does not involve data collected from social media platforms.

## CRediT Author Statement

**Ahmad K. Sleiti:** Conceptualization, Methodology, Software, Data curation, Writing- Original draft preparation, Visualization, Investigation, Validation, Writing- Reviewing and Editing.

## Declaration of Competing Interest

The authors declare that they have no known competing financial interests or personal relationships that could have appeared to influence the work reported in this paper.
